# Assessment of α-Synuclein Secretion in Mouse and Human Brain Parenchyma

**DOI:** 10.1371/journal.pone.0022225

**Published:** 2011-07-14

**Authors:** Evangelia Emmanouilidou, Dimitris Elenis, Themis Papasilekas, Georgios Stranjalis, Kyriaki Gerozissis, Penelopi C. Ioannou, Kostas Vekrellis

**Affiliations:** 1 Division of Basic Neurosciences, Biomedical Research Foundation of the Academy of Athens, Athens, Greece; 2 Department of Chemistry, University of Athens, Athens, Greece; 3 Department of Neurosurgery, Evaggelismos General Hospital, University of Athens, Athens, Greece; 4 CNRS, Center of Neurosciences, Paris-Sud, UMR 8195, Orsay, France; 5 University Paris-Sud, UMR 8195, Orsay, France; 6 INSERM, Orsay, France; National Institutes of Health, United States of America

## Abstract

Genetic, biochemical, and animal model studies strongly suggest a central role for α-synuclein in the pathogenesis of Parkinson's disease. α-synuclein lacks a signal peptide sequence and has thus been considered a cytosolic protein. Recent data has suggested that the protein may be released from cells via a non-classical secretory pathway and may therefore exert paracrine effects in the extracellular environment. However, proof that α-synuclein is actually secreted into the brain extracellular space in vivo has not been obtained. We developed a novel highly sensitive ELISA in conjugation with an in vivo microdialysis technique to measure α-synuclein in brain interstitial fluid. We show for the first time that α-synuclein is readily detected in the interstitial fluid of both α-synuclein transgenic mice and human patients with traumatic brain injury. Our data suggest that α-synuclein is physiologically secreted by neurons in vivo. This interstitial fluid pool of the protein may have a role in the propagation of synuclein pathology and progression of Parkinson's disease.

## Introduction

α-Synuclein is linked genetically and biochemically to Parkinson's Disease (PD) [1]. α-Synuclein is the principal constituent of proteinaceous inclusions termed Lewy Bodies, the pathological hallmark of PD [2]. Point mutations as well as multiplications in the locus encoding for α-synuclein are linked with familial cases of PD [3], [4], [5]. The mechanism of involvement of α-synuclein in PD is not well understood, although there is evidence that abnormal folding plays a critical role in the pathogenesis of the disease and that the toxic α-synuclein species may be oligomeric intermediates [6], [7]. Until recently, α-synuclein was considered to exert its pathogenic effects in the cytoplasm of the cells. However, it was demonstrated that soluble monomeric and oligomeric forms of α-synuclein are present in human cerebrospinal fluid (CSF) and blood plasma of healthy and diseased individuals. [8], [9]. These results suggest that the physiological as well as the aberrant actions of α-synuclein can extend to the extracellular space and neighboring cells. However, the species and mechanisms involved in such actions have not been elucidated.

We recently showed that α-synuclein can be physiologically released to the extracellular space of overexpressing cells in a calcium dependent manner and partly via a mechanism that involves exosomes [10]. Extracellular α-synuclein can be toxic to recipient neurons [10], [11]. In support for a pathogenic role for extracellular α-synuclein, recent reports show that α-synuclein aggregates released from neuronal cells can be transferred to neighboring neurons forming Lewy-like inclusions [12], providing mechanistic basis for the development of Lewy pathology in normal mesencephalic transplants in PD patients [13], [14]. To this end, recent data demonstrated *in vivo* transfer of α-synuclein to dopaminergic neurons grafted to the striatum of transgenic mice overexpressing human α-synuclein [15] bolstering the hypothesis of a cell-to-cell spread of α-synuclein pathology. Interestingly, Danzer et al., recently demonstrated the uptake of secreted α-synuclein oligomers by neurons and their detrimental effect on neuronal survival [16].

Still, despite these exciting findings, the evidence for extracellular α-synuclein in the brain has been controversial and not yet fully verified and relies primarily on its presence in human CSF. It is therefore crucial to examine whether α-synuclein is actually released at biologically meaningful concentrations in an *in vivo* context and in the absence of any evidence of cell death. Understanding the mechanisms which underlie the regulation of α-synuclein secretion in normal physiology may provide unique insights into new unidentified factors in the progression and/or pathogenesis of PD.

In the current study, we have developed a novel highly sensitive ELISA and employed microdialysis to investigate the presence of α-synuclein in the interstitial fluid (ISF) of awake adult A53T α-synuclein transgenic mice [17] that develop α-synuclein oligomerization and in human subjects with severe brain injury who undergo microdialysis. Our data show that α-synuclein is normally present in the brain parenchyma. As such, it may have as yet unidentified physiological and pathogenic roles in the extracellular space.

## Methods

### Animals

For *in vivo* microdialysis experiments we used male homozygous transgenic (Tg) C57BI/C3H mice expressing human A53T α-synuclein under the control of the prion promoter (Jackson Laboratory, Bar Harbor, Main). The generation and phenotype of these mice has been previously described [17]. Mice were used at 8–12 months of age. Wild type (WT) littermates and C57BL6/JOlaHsd synuclein null mice (stain number Harlan Laboratories) of the same sex and age were used as controls to verify signal specificity [18]. Animals were housed in the animal facility of the Biomedical Research Foundation of the Academy of Athens (BRFAA) in a room with a controlled light-dark cycle (12 hours light-12 hours dark) and free access to food and water.

### In vitro microdialysis

In vitro microdialysis experiments were performed in human CSF. For *in vitro* recovery, low molecular weight secreted α-synuclein species from SHSY5Y overexpressing the WT form of the protein were also used as an alternative source of α-synuclein (prepared as described in [10]). In some experiments, CSF was spiked with recombinant α-synuclein (gift from H. A. Lashuel, EPFL, Lausanne, Switzerland). Lyophilized α-synuclein was reconstituted in PBS, filtered through a 0.2 µm filter and then through a 100 kDa cut off filter (Millipore) to yield pure monomeric protein.

The probes used for mouse microdialysis (CMA-12 custom made, 2 mm length, 0.5 mm diameter, 100 kDa cut-off) were connected to a CMA 402 syringe pump through teflon (FEP) tubing (inner diameter 0.12 mm; 1.2 µl/100 mm, CMA). The probes used for human microdialysis (CMA 71, 30 mm length, 0.5 mm diameter, 100 kDa cut-off) were connected to a CMA 106 syringe pump (fixed flow rate 0.3 µl/min). Probes were first washed with artificial CSF (CNS perfusion fluid, CMA) containing 0.15% BSA (Sigma) previously filtered through a 100 kDa cut off filter (Millipore).

### In vivo mice microdialysis

Guide cannulas were stereotaxically implanted in the striatum under isoflurane anesthesia (4–2.5%) as previously described [19]. Animals were kept anesthetized during the whole procedure. Breathing was kept stable using an oxygen / air ratio of 0.5. Bore holes were made above the right striatum according to the mouse atlas of Paxinos and Franklin (coordinates, AP = +0.5 mm, ML = −2.2 mm, DV = −2.4 mm). CMA 12 guide cannulas were inserted and fixed to the skull with stainless steel screws and dental cement. Mice were removed from the stereotaxic device and allowed to recover in individual cages. 72–96 hrs after surgery, mice were moved to the microdialysis cage. During microdialysis, mice were awake and had free access to food and water (CMA 120 System for Freely Moving Animals). CMA 12 custom made probes were manually inserted and connected to the CMA 402 syringe pump with a constant flow rate of 0.6 µl/min. Prior to sample collection, the probe was allowed to equilibrate for at least 2 hrs with the same flow rate. Samples were collected bihourly for 6 hrs using a CMA 170 refrigerated fraction collector and stored at −80°C until analyzed by ELISA. At the end of the experiment, the brain was excised, fixed in 4% paraformaldehyde at 4°C and analyzed for probe placement with 2% Coomassie blue staining. All efforts were made to minimize animal suffering and to reduce the number of the animals used, according to the European Communities Council Directive (86/609/EEC) guidelines for the care and use of laboratory animals. All animal experiments were approved by the Institutional Animal Care and Use Committee of BRFAA (permit number A.05.1/6/02-07).

### Histochemical processing

Mice were deeply anesthetized by an overdose of pentobarbital and perfused transcardially first with 30 ml PBS and then with 30 ml of ice-cold 4% paraformaldehyde in PBS. Brains were quickly removed, post-fixed in the same fixative for 16 hours at 4°C and cryoprotected first in 15% sucrose in PBS for 24 h and then in 30% sucrose in PBS for 24 h at 4°C. Finally, brains were frozen at −45°C and stored at −80°C until sectioning. For each mouse, free-floating cryostat-cut sections (30 µm) were collected using a Bright cryostat at −25°C at the levels of striatum (AP, 0.2 mm from bregma).

### Tissue double labeling

For double-fluorescence labelling, 30 µm free-floating sections were rinsed in three changes of PBS for 5 min each and then blocked for 60 min in 2% NGS in PBS containing 0.1% Triton-X (blocking buffer). Sections were then incubated with anti-NeuN antibody (mouse IgG1, 1∶250, Millipore) and anti-tyrosine hydroxylase (TH) antibody (rabbit IgG, 1∶1000, Chemicon) in blocking buffer for 16 hours at 4°C. Sections were again washed as above and then transferred to a mixture of Cy3-conjugated anti-mouse and Cy2 secondary–conjugated anti-rabbit antibodies (Jackson ImmunoResearch), each diluted 1∶100 in blocking buffer, for 60 min at room temperature. Sections were first rinsed three times in PBS for 5 min each, then in H_2_O for 2 min, and finally sections were mounted on Superfrost plus slides (VWR) and air-dried for 16 hours at 37°C. For Fluoro-Jade C staining, NeuN-stained sections were rehydrated for 2 min in H_2_O and then transferred to a 0.06% potassium permanganate solution for 20 min. Sections were rinsed once more in H_2_O for 2 min and transferred to 0.0002% Fluoro-Jade C in 0.1% acetic acid for 10 min. Following staining, slides were washed three times in H_2_O, air-dried for 16 hours at 37°C, xylene-cleared and coverslipped with DPX. Images were obtained in either a Leica DMRA2 upright microscope or in a Leica SP5-II confocal microscope.

### Human CSF samples

CSF samples were obtained from Normal Pressure Hydrocephalus patients undergoing ventriculoperitoneal shunting in Evaggelismos Hospital (Athens, Greece). All subjects were otherwise healthy and in a good general condition. CSF was collected upon insertion of the ventricular catheter (initial flow discarded to avoid blood contamination). The study was approved by the Evaggelismos Hospital Bioethics Board. CSF collection was carried out with the informed written consent of all patients.

### In vivo human microdialysis

Human microdialysis samples were obtained from patients (male and female; 30–60 years old) suffering from severe head injury (Glascow Coma Scale ≤8 following cardiopulmonary resuscitation) and admitted to the ICU. All patients had an abnormal admitting CT scan and microdialysis was part of their routine monitoring. Their past history was unremarkable and coagulation normal. Approval for using the microdialysis samples in the current study was obtained from the Evaggelismos Hospital Bioethics Board following the principles expressed in the Declaration of Helsinki.

CMA 71 microdialysis catheters (30 mm, 0.5 mm diameter, 100 kDa cut-off) were placed into the non-lesioned cerebral cortex and, whenever possible, to the right frontal lobe. Insertion was via a cranial access device and through a single burr-hole placed on Kocher's point (2–3 cm off the midline; to avoid the superior sagital sinuson, 1 cm in front of the coronal suture; to avoid the motor cortex). Catheters were connected to the CMA 106 syringe pump and perfused with CMA CNS perfusion fluid at a flow rate of 0.3 µl/min. Microdialysis vials were changed every 120 min. The duration of microdialysis sampling was at least 72 hours.

Samples obtained the first 12 hours of patient monitoring were excluded from the analysis to eliminate the insertion artifact.

### Ultra-sensitive ELISA for α-synuclein

For the sandwich ELISA, the monoclonal Syn-1 antibody (BD Biosciences), raised against amino acids 15–123 of the human, mouse or rat α-synuclein sequence, was used as capture antibody. This antibody recognizes a conserved epitope in human and rodent α-synuclein (residues 91–99) whereas it shows no reactivity for the β- or γ-synuclein isoforms [20]. The polyclonal C-20 antibody (Santa Cruz), raised against a C-terminus peptide of human α-synuclein, was used for antigen detection through direct conjugation with HRP (Pierce). Each ELISA plate (Corning Costar) was coated for 24 hrs at room temperature with 0.5 µg/ml of Syn-1 (50 µl per well) in 100 mM NaHCO_3_, pH 9.3. Following coating, plates were stored at 4°C for up to 2–3 weeks. The plates were washed three times in wash buffer (50 mM Tris-HCl, 150 mM NaCl and 0.04% Tween-20) and 50 µl of microdialysis sample or recombinant α-synuclein (as standard), appropriately diluted in TBST/BSA (10 mM Tris-Cl, pH 7.6, 100 mM NaCl, 0.1% Tween-20 and 1% BSA) was added. To allow antigen binding, plates were incubated at 37°C for 2 ½ hrs. After washing three times with wash buffer, 50 µl of HRP-conjugated C-20 antibody (2500x diluted in TBST/BSA) were added to each well and further incubated for 1 hr at ambient temperature. The wells were washed and 50 µl of chemiluminogenic HRP substrate (ultrasensitive luminol reagent, BioFX Laboratories) were added to each well. The wells were incubated for 15 min at room temperature and the chemiluminescence was integrated for 1 s.

### Immunodepletion of α-synuclein

Human microdialysis samples or human CSF were immunodepleted of α-synuclein by immunoprecipitation with 1 µg of Syn-1, mouse monoclonal antibody (BD Biosciences) as previously described [10].

### Western blotting

Western blotting of striatum homogenates was performed as previously described [21]. Immunoblotting was performed using Syn-1 monoclonal anti-α-synuclein antibody (BD Biosciences). Differences in protein expression levels were quantified using Gel Analyser software after standardization of all values with β-actin (monoclonal antibody, Sigma) as loading control. Statistical analysis was performed using the Student's t-test, *p* values of <0.05 were considered significant.

## Results and Discussion

There has been increasing amount of evidence suggesting that α-synuclein, a protein with mainly cytosolic localization, can be detected in the plasma and cerebrospinal fluid (CSF) of humans and in the culture media of neuronal cells [8], [10], [22], [23]. However, it is generally accepted that the main fraction of proteins detected in the normal CSF originates from blood and only the 20% of CSF proteins are brain-derived [24]. Red blood cells have been identified as the major source of α-synuclein in the blood [25]. Considering the abundance and fragility of these cells, α-synuclein quantitation in other body fluids, such as CSF, may be compromised by contamination with intact or lysed red blood cells. The fundamental aim of the current study was to assess *in vivo* the dynamics of extracellular α-synuclein concentration in the location of its release, the brain. For that purpose, we used *in vivo* microdialysis to investigate whether α-synuclein is present in the ISF of WT and Tg mice that overexpress the A53T mutant form of α-synuclein (A53T Tg). Two basic advantages of this technique make it most appropriate for this investigation. First, the microdialysis samples are devoid of potential substances or degradation enzymes that could interfere with the final measurement of the target protein. Second, unlike CSF, changes of the peptide of interest in the microdialysis samples do not follow/reflect changes of the peptide in plasma [26], [27].

**Figure 1:**
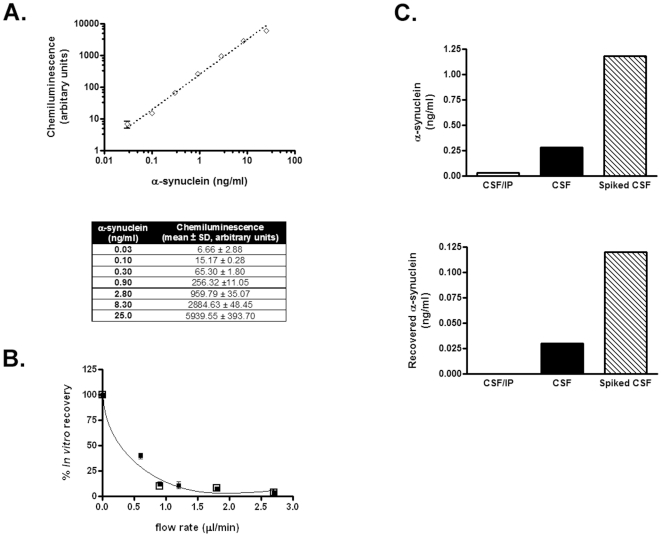
In vitro microdialysis to measure α-synuclein. (A) Calibration curve for a novel in-house ELISA for α-synuclein. Serial dilutions of recombinant human WT α-synuclein were loaded in quadruplicate and read with the new ELISA. Each point represents the mean value measured for each concentration (n = 4, mean ± SD) as shown in the corresponding table. (B) *In vitro* percentage recoveries for α-synuclein using the interpolated zero flow method. Following microdialysis, α-synuclein was measured at various flow rates in human CSF (filled squares) or low molecular weight secreted α-synuclein species (open squares). The plot is representative of three individual experiments each corresponding to different CSF samples or low molecular weight preparations. (C) Microdialysis reflects changes in α-synuclein concentration in the sample. Human CSF was either immunodepleted from α-synuclein (CSF/IP) or spiked with 4 ng/ml of recombinant α-synuclein (Spiked CSF). α-synuclein concentration was determined by ELISA in the CSF samples (right panel) and the microdialysates (left panel). Immunodepletion of α-synuclein from the CSF showed only baseline levels of α-synuclein. Spiking with exogenous α-synuclein resulted in a 4.2-fold increase in α-synuclein concentration in the CSF sample and in a 4.0-fold increase in the microdialysates. The graph represents one of three similar experiments.

The measurement of the concentration of substances in the extracellular space of the human brain, including macromolecules, using microdialysis is now well established [28], [29], [30], [31], [32]. However, analyzing proteins in microdialysis samples has been challenging due to the low concentration of the target protein in the sample of interest and the small amount of ISF often available [14]. To address this issue, we have developed a new, ultra-sensitive ELISA to determine α-synuclein concentration in biological samples, including plasma, CSF and ISF. The detection of the assay is based on HRP-conjugated α-synuclein-specific (C-20) antibody for the rapid chemiluminometric determination of α-synuclein, which greatly increases the detectability and reproducibility of the assay. The detection limit and linear range of the assay was established by analyzing serial dilutions of recombinant human α-synuclein (0.03, 0.1, 0.3, 0.9, 2.8, 8.3, and 25 ng/ml) (Fig. 1 A). The limit of quantification (LOQ) was less than 0.01 ng/ml (defined as the concentration with a signal/background ratio of 2). The analytical range of the assay extends from 0.01 up to 25 ng/ml.

Our results show that our new ELISA can be used for the detection of full length α-synuclein with high sensitivity and specificity. It should be noted that, based on the antibodies selected, C-terminal truncated or oligomeric forms of α-synuclein would not be captured by this method. In an attempt to assess the applicability of α-synuclein as a biomarker for PD, various ELISA systems have been developed for the determination of α-synuclein concentration in biological fluids [33], [34], [35], some of which being oligomer-specific [9], [22]. Even though these methods provide high specificity and accuracy, there is great variability in the amount of α-synuclein quantified in either blood plasma or CSF. The different ELISA systems employed (in terms of the antibodies and the detection method used) and the different protocols for sample collection and processing probably account for such discrepancies. The α-synuclein specific ELISA developed in this work offers higher sensitivity compared with previously described methods. The analytical performance and the application of this method for the quantification of α-synuclein in biological fluids (CSF and plasma) will be described in detail in another study (Emmanouilidou et al, manuscript in preparation).

To ensure α-synuclein detection following microdialysis in mice, we used A53T Tg mice which, as demonstrated by western blot and densitometry analysis, exhibit a 3-fold increase in α-synuclein levels in the striatum compared to WT littermates (2.8±1.2 fold increase, n = 4) (Figure S1). All mice were used at 8–12 months of age. In our hands, at this age, A53T Tg mice show no significant variation in the striatal α-synuclein levels, no development of any motor phenotype and no abnormalities in the dopaminergic system [21].

α-Synuclein is a 140 aa protein with a molecular weight of ∼14 kDa under denaturing conditions. Being a natively unfolded protein, α-synuclein has been sometimes found to exhibit an anomalous molecular weight of 50–60 kDa under certain native conditions [36], [37], [38]. We, therefore, chose to perform α-synuclein microdialysis studies *in vitro* using a custom made probe with a 100 kDa cut off membrane. During microdialysis, a physiologically compatible perfusion fluid is delivered through the probe at a low and constant flow rate so that diffusion of solutes occurs in both directions across the semi-permeable membrane of the probe. In the case that the perfusion fluid does not contain the molecule of interest, the concentration in the microdialysate represents a fraction of the tissue diffusible levels. This is referred to as the relative recovery [39]. We used the interpolated zero flow method [19] to define the percentage recovery of α-synuclein through the 100 kDa probe in human CSF or in a solution containing low molecular weight species of cell secreted α-synuclein (Fig. 1 B). The mean % recoveries of α-synuclein were similar in these two solutions at room temperature, achieving a maximum (∼40%) at a microdialysis flow rate of 0.6 µl/min, the lowest rate we tested (Fig. 1 B). We further assessed whether changes in the concentration of α-synuclein in the microdialysate directly reflect changes in the α-synuclein concentration in the sample of interest. To this end, we performed *in vitro* microdialysis on human CSF before and after spiking with recombinant α-synuclein (Fig. 1 C). α-Synuclein concentration was estimated by our new ELISA in the starting CSF samples and in the resultant microdialysates. The increase in α-synuclein concentration by spiking with exogenous α-synuclein was 4.2-fold for the CSF samples and 4.0-fold for the microdialysates. Further demonstrating that microdialysis monitors changes in α-synuclein concentration in the solution, α-synuclein immunodepletion of the CSF using Syn-1 antibody [10] resulted in barely detectable α-synuclein levels (Fig. 1 C).

**Figure 2:**
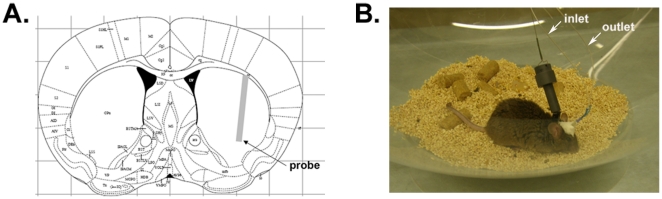
Probe implantation within mouse striatum. (A) Schematic illustration showing the stereotactic placement of a 2 mm-probe in mouse striatum according to the mouse atlas of Paxinos and Franklin. (B) Photograph of awake, freely moving mouse following insertion of guide cannula and microdialysis probe.

**Figure 3:**
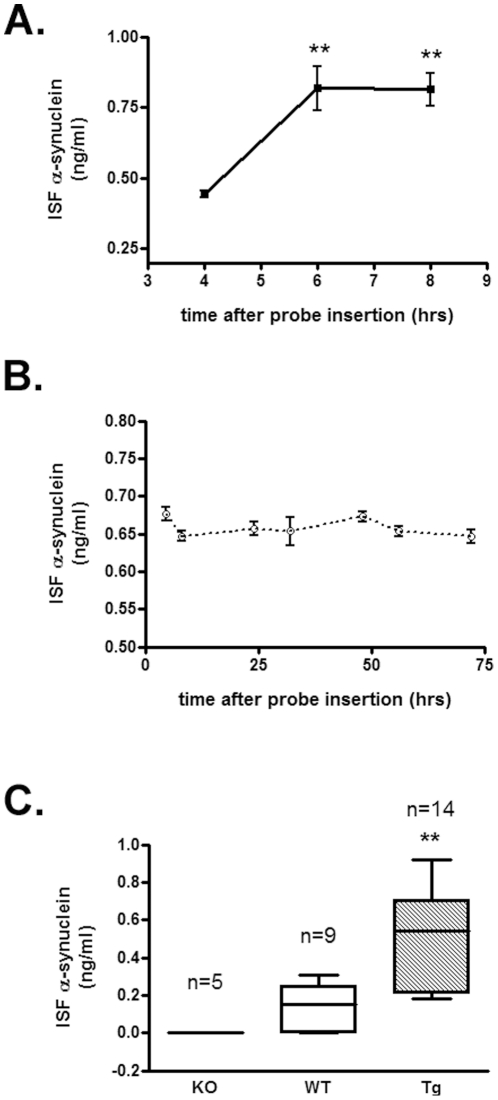
In vivo microdialysis in mouse ISF. (A) α-Synuclein concentration in ISF of Tg mice is stable 6 hours after probe implantation. Probe was allowed to equilibrate in mouse striatum for 2 hours and three microdialysate samples were collected at 2 hour-intervals at a flow rate of 0.6 µl/min. α-Synuclein concentration in the microdialysate samples was determined by ELISA. Stable levels of α-synuclein were recovered only 6 hours following probe insertion (n = 3, mean ± SD, one way ANOVA test followed by Tukey's test, ^**^ *p*<0.01). (B) The levels of α-synuclein in mouse ISF remain constant over a period of 3 days. Probe was allowed to equilibrate for 4 hours before fraction collection. α-Synuclein concentration in the microdialysates was measured by ELISA. (C) *In vivo* concentration of α-synuclein in the ISF of knock-out (KO), wild type (WT) and transgenic (Tg) mice. Microdialysis was performed as described in the Methods section. α-Synuclein concentration was determined by ELISA 6 hours following probe insertion. α-Synuclein levels were significantly increased in Tg mice (0.49±0.27 ng/ml, n = 14) compared to the WT mice (0.15±0.12 ng/ml, n = 9). Data are presented as mean ± SD and statistics were performed by one way ANOVA test followed by Tukey's test (^**^ *p*<0.01). As expected, α-synuclein was not detected in the microdialysates of KO mice (n = 5).

After validation of α-synuclein measurement by microdialysis *in vitro*, we assessed measurements of α-synuclein in the ISF of WT and A53T Tg mice *in vivo*. Guide cannulas were implanted stereotactically under isoflurane anaesthesia, and 100 kDa cut-off probes were inserted into the striatum (Fig. 2). Correct probe placement was verified by Coomassie staining (Figure S2). Probe insertion may cause acute local injury of the brain area adjacent to the probe tip, leading to a local compromise of blood brain barrier integrity. However, due to rapid repair mechanisms, the blood brain barrier appears to be largely impermeable to relatively large molecules, such as albumin-bound Evan's blue dye, within only 30 min of probe placement [40]. Chronic damage, marked by astrogliosis and inflammation, has been shown to begin 24–36 hours after probe implantation [19]. To avoid artifacts originating from local tissue injury, our α-synuclein measurements in mouse ISF were restricted to a window of 4–8 hrs after probe insertion. ISF α-synuclein concentration reached stable levels at 6 hours after probe insertion and remained stable even after 72 hours following probe placement (Fig. 3 A, B). Further demonstrating that the measured α-synuclein concentration reflects the presence of the protein in the mouse ISF, and not an artifact of plasma membrane leakage, the median α-synuclein at 6 and 8 hours was 46% higher than at 4 hours after probe insertion (comparison performed by one way ANOVA test followed by Tukey's test, *p* = 0.01) (Fig. 3 A). This result is important considering that, if α-synuclein were released due to neuronal membrane damage following probe placement, we would have expected to detect high initial concentrations of α-synuclein after probe insertion (2–4 hours) followed by a gradual decrease at later time points, but we see the opposite.

We performed *in vivo* microdialysis in male WT and A53T Tg mice with a flow rate of 0.6 µl/min. Two-hour fractions of microdialysate were collected over a period of 4–8 hours following probe insertion, and measured by ELISA. Figure 3 C summarizes the mean α-synuclein concentrations in the ISF of these mice 6 hours after probe insertion. The mean ISF α-synuclein concentration was 0.15±0.12 ng/ml for WT (n = 9) and 0.49±0.27 ng/ml for A53T Tg animals (n = 14). Our ELISA detected no α-synuclein in the ISF microdialysates of α-synuclein knock-out mice analyzed in the same manner, strongly validating the specificity of our method (n = 5) (Fig. 3 C). In agreement with α-synuclein levels determined in the whole striatum by western blotting (Figure S1) Tg animals also demonstrate a ∼3 fold increase in the ISF α-synuclein concentration compared with WT animals. Taken together, our *in vivo* microdialysis data suggest that α-synuclein is physiologically present in the mouse brain parenchyma.

**Figure 4:**
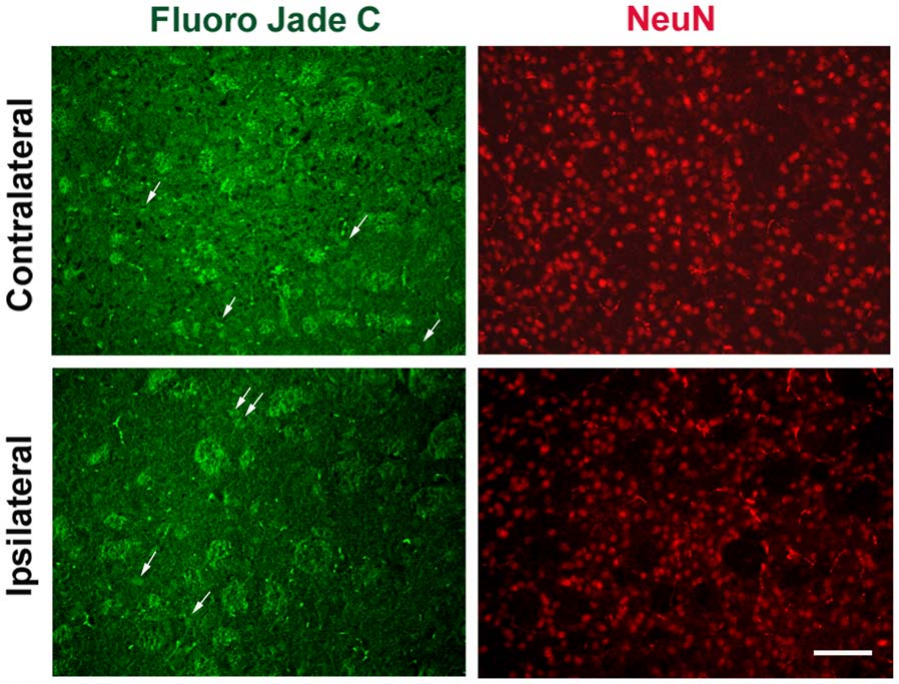
Probe insertion does not cause significant cell loss in mouse striatum. Coronal striatal sections obtained after microdialysis stained for the presence of degenerating neurons with the fluorescent dye, Fluoro-jade c (A and C). Arrows indicate degenerating neuronal nuclei at the guide cannula insertion side (ipsilateral side) and the opposite, non-treated side (contralateral side). Sections were co-stained with NeuN (C and D) to mark neuronal nuclei. Images were obtained with fluorescence microscope under 20x magnification, scale bar, 50 µm.

**Figure 5:**
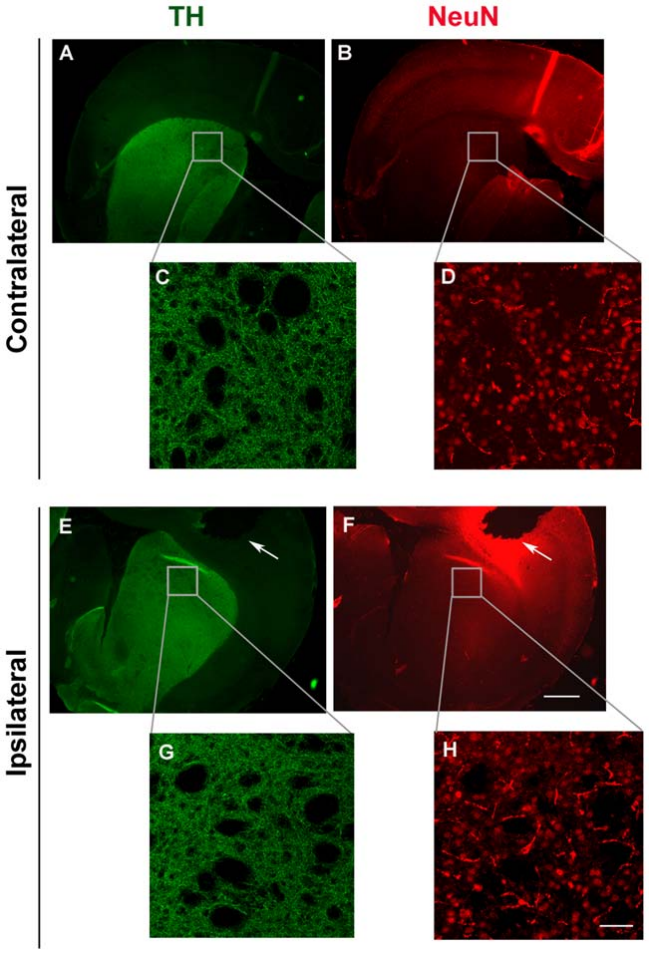
Striatal neuronal fiber density is not affected by probe placement. Representative coronal sections showing the side of probe insertion (ipsilateral side, E-H) and the opposite, control side (contralateral side, A–D). Sections were double stained with TH (green) and NeuN (red) and visualized under low 2.5x magnification (A, B, E, F, scale bar, 1 mm). Confocal images C, D, G, and H represent enlargement of the areas in boxes (40x magnification, scale bar, 50 µm). White arrows indicate tissue damage caused by guide cannula implantation.

It has been shown that erythrocytes are the major source of α-synuclein in blood [25]. In addition, probe insertion could cause local tissue damage in the brain leading to cell death. To exclude the possibility that α-synuclein detected in mouse ISF was an overestimation due to blood contamination or cellular damage, we initially measured α-synuclein in the plasma of mice that had been subjected to microdialysis. The levels of α-synuclein in mouse plasma (∼3 ng/ml) were at least 6-fold higher than those found in mouse ISF. Importantly, we found that α-synuclein levels in ISF remained constant over a period of 3 days and did not decline over time (Fig. 3 C). Thus, it seems unlikely that our α-synuclein readings in mouse ISF originate from plasma oozing into the brain. In addition, we assessed neuronal degeneration in mouse striatum after microdialysis using the fluorescent dye, Fluoro-Jade C [41], [42]. Coronal brain sections were double stained with the Fluoro-Jade C dye, which labels degenerating neurons, and anti-NeuN antibody which labels the total number of neuronal nuclei. We found no significant difference in neuronal degeneration in the striatum comparing the site of probe insertion (ipsilateral site) with the normal site (contralateral site) (Fig. 4). To this end, we also assessed the integrity of dopaminergic neuronal fibres in the striatum following probe implantation. Coronal sections were double labelled with anti-TH and anti-NeuN antibodies and TH density was analysed in the ipsilateral and the contralateral sites using fluorescence microscopy. Local tissue damage caused by guide cannula implantation was evident in the cortex. However, striatal TH fiber density was not greatly affected (Fig. 5) suggesting that probe insertion does not cause significant lesion in the area where microdialysis sampling is performed.

**Figure 6:**
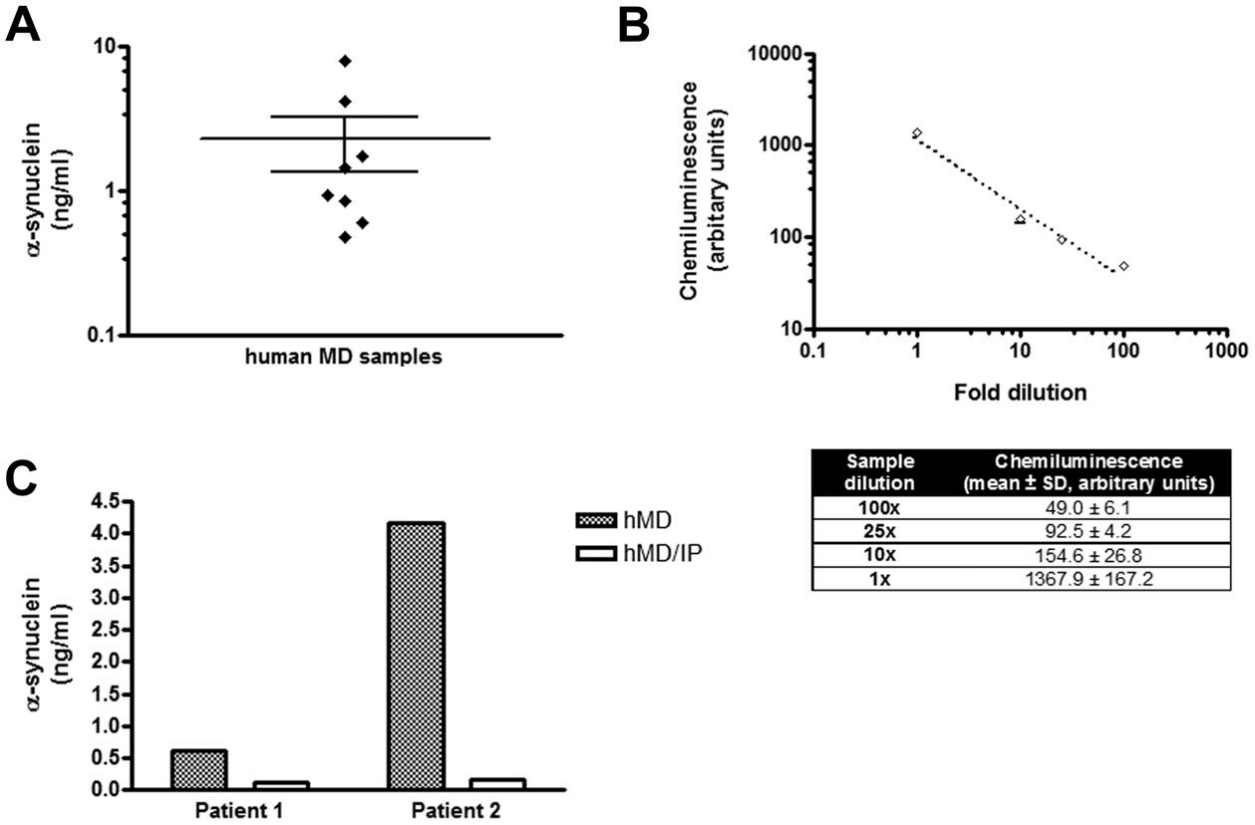
α-Synuclein is present in human brain parenchyma. (A) Brain ISF α-synuclein concentrations of individual patients. Human microdialysis was performed as described in the Methods section and microdialysate samples were assayed by ELISA for the presence of α-synuclein. Each dot in the plot represents the ISF α-synuclein concentration of each patient (n = 8, data presented as the mean of two randomly picked microdialysate samples). (B) Signal specificity in the human microdialysis samples. Serial dilutions of a human microdialysate were measured by ELISA. The chemiluminescence values obtained were linearly correlated to the dilution factor of the sample. Data are presented as mean ± SD (n = 3) and are shown in detail in the corresponding table. (C) Immunodepletion of α-synuclein from human microdialysates decreases α-synuclein to the basal levels. Microdialysates (hMD) from two patients were separately pooled and immunoprecipitated with α-synuclein antibody (hMD/IP). For each patient, control and immunodepleted samples were measured using the ELISA for α-synuclein.

To further establish the physiological significance of our findings in mice, we went on to examine whether α-synuclein is present in human brain parenchyma. *In vivo* intracerebral microdialysis has been well established as a bedside technique for clinical neuroscience providing important information on several neurological conditions such as traumatic brain injury [43], [44] and seizures [45]. Importantly, intracerebral microdialysis has previously been used for monitoring amyloid β protein and tau levels as a marker of axonal injury in humans [30], [31]. We analyzed ISF samples from 8 patients with severe brain injury who were admitted to the ICU and had intracranial monitoring for clinical purposes. The microdialysis probe was left in situ for 72 hours to minimize the risk of infection. Microdialysis sampling in human brain have also been reported for periods up to 6 days with no reference of side effects due to inflammation [29], [44], [46]. Two microdialysis samples from each patient were randomly selected from 24 to 36 hours after probe insertion and assayed for α-synuclein with our new ELISA. α-Synuclein was readily detected in the ISF of all patients (Fig. 6 A). We next performed *in vitro* microdialysis to estimate the % recovery of α-synuclein through the 100 kDa CMA 71 probe that was used to obtain the human microdialysates. Using the interpolated zero flux method [19], the % recovery of α-synuclein in human CSF spiked with known concentrations of recombinant α-synuclein (2–16 ng/ml) was found to be ∼80% at a flow rate of 0.3 µl/min (data not shown). After correction for this % recovery, α-synuclein concentrations in human ISF varied from 0.5 to 8.0 ng/ml (n = 8). None of the patients analysed had a diagnosis of PD or other dementia, suggesting that the presence of α-synuclein in human brain parenchyma is not disease-related. Although the probe was inserted in a healthy brain area, away from the injured side, one cannot rule out the possibility that α-synuclein levels we measured in the human ISF are not affected by the overall brain status. Various parameters, such as synaptic activity and metabolism, may affect the levels of extracellular α-synuclein in humans. In this sense, the values obtained in our study do not represent control reference measurements of α-synuclein but rather prove the presence of the protein in the human brain.

To verify that the ELISA values obtained from human ISF correspond to true α-synuclein concentrations, we performed two sets of experiments. First, serial dilutions of a human ISF microdialysate having a relatively high α-synuclein concentration were prepared and assayed by ELISA. As expected, the chemiluminescence obtained from each dilution was linearly correlated with the dilution factor of the sample (Fig. 6 B). Second, human microdialysates from two patients were immunodepleted of α-synuclein using the Syn-1 antibody [10]. α-Synuclein levels were compared before and after immunodepletion. This resulted in almost undetectable levels of α-synuclein (Fig. 6 C). Taken together, these experiments indicate that our ELISA readings represent changes in the amount of α-synuclein in human ISF collected by *in vivo* microdialysis.

In conclusion, our data suggests that α-synuclein is physiologically released into the interstitial fluid of the brain in mice and humans, and they further suggest as role for secreted α-synuclein under physiological and pathologic conditions. It is clear that α-synuclein is a key molecule in familial and sporadic PD. Most notably, relatively small (50%) increases in its levels are sufficient to cause PD in humans, and it accumulates early in the brains of patients with sporadic disease. This study demonstrates direct evidence that soluble, ISF α-synuclein can be measured in brain parenchyma *in vivo*. This secretable form of α-synuclein may be biologically important, since it could exert paracrine effects on neighboring cells. The physiological role for extracellular α-synuclein remains to be identified. Maintenance of the intracellular steady state concentration of α-synuclein is considered a key challenge for neuronal homeostasis, and elevated total brain levels of the protein have been directly linked with PD pathogenesis. It is possible that a dynamic equilibrium between intracellular and extracellular α-synuclein may exist ensuring normal functioning of neuronal cells. In this respect, dysfunctions in the mechanism(s) regulating extracellular α-synuclein levels, such as mechanisms of secretion, re-uptake or extracellular clearance, may affect neuronal survival. Increases in extracellular α-synuclein could trigger toxic oligomer formation and result in inflammatory glial activation [47], finally leading to neurodegeneration. However, any oligomer formation might be expected to occur in the concentrated milieu of the cytoplasm rather than in the more dilute extracellular fluid, and it will now be important to ascertain whether soluble oligomers are also released *in vivo*. According to Braak [48], the progression of PD symptoms could be due to spreading of misfolded α-synuclein along poorly myelinated axonal pathways. It was recently shown in postmortem studies that embryonic stem cells grafted into the brains of people with PD exhibited Lewy body pathology and α-synuclein accumulation [13], [14]. Furthermore, Hansen et al. recently demonstrated that α-synuclein transmission *in vivo* depends on endocytosis [15]. These findings suggest the possibility of an α-synuclein host-to-graft propagation process in PD. The existence of secreted α-synuclein in the living brain as validated by our work supports this hypothesis. Much cell biological work will now be required to elucidate the non-classical secretory mechanism(s) of α-synuclein *in vivo*. We believe that our approach will allow the systematic investigation of these mechanisms and also provide insights to understanding PD pathogenesis. In this respect, down-regulation of extracellular α-synuclein levels could be a potential target for the development of treatment strategies for PD.

## Supporting Information

Figure S1 **α-synuclein levels in the striatum of WT and A53T Tg mice.** Representative immunoblot of striatum homogenates from WT and A53T Tg mice (n = 4) analyzed for the presence of α-synuclein using the Syn-1 antibody. β-actin is used as loading control. Quantitative densitometric analysis (right panel) demonstrates a 2.8±1.2 fold increase in α-synuclein striatum levels of Tg mice compared with WT mice (n = 4, mean ± SD, independent t-test, *p<0.05).
(DOC)

Figure S2 **Probe placement in the mouse striatum.** Representative image showing probe placement in mouse striatum according to the mouse atlas of Paxinos and Franklin. Intense staining depicts location (arrows) of the probe membrane through the area of striatum.
(DOC)
