# Supplementary figures and images for: Assessment of α-Synuclein Secretion in Mouse and Human Brain Parenchyma
Source: PLoS One. 2011 Jul 14;6(7):e22225. doi: 10.1371/journal.pone.0022225 (PMC3136497; doi:10.1371/journal.pone.0022225)

**Supplementary Figure 1**


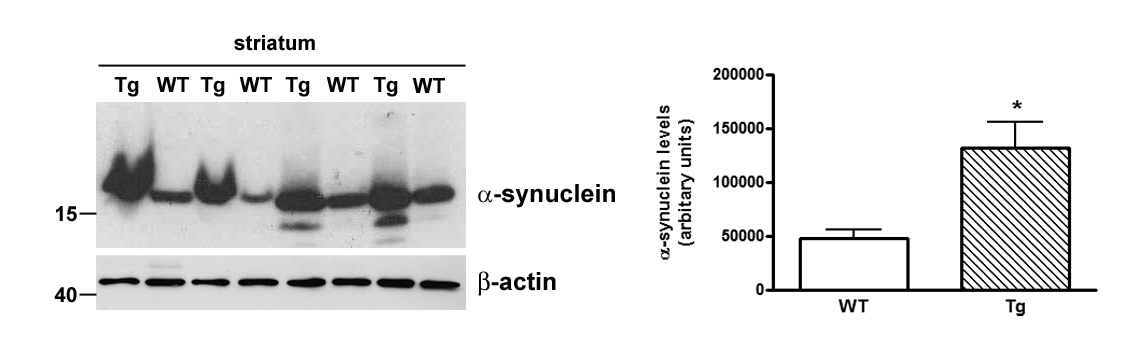

Supplement: Figure S1 — α-synuclein levels in the striatum of WT and A53T Tg mice. Representative immunoblot of striatum homogenates from WT and A53T Tg mice (n = 4) analyzed for the presence of α-synuclein using the Syn-1 antibody. β-actin is used as loading control. Quantitative densitometric analysis (right panel) demonstrates a 2.8±1.2 fold increase in α-synuclein striatum levels of Tg mice compared with WT mice (n = 4, mean ± SD, independent t-test, *p<0.05). (DOC) [file pone.0022225.s001.doc]

**Supplementary Figure 2**


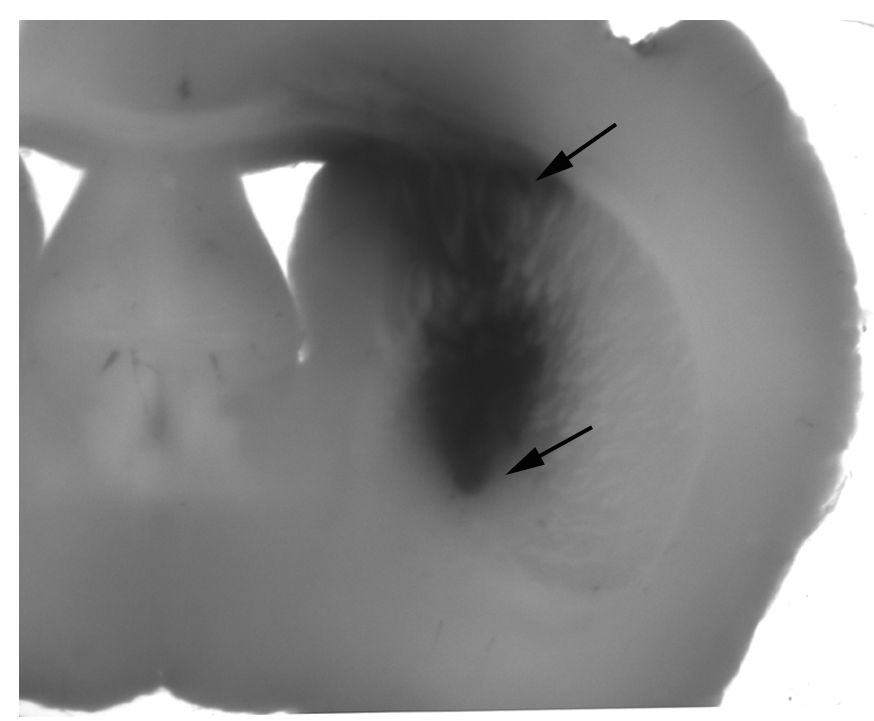

Supplement: Figure S2 — Probe placement in the mouse striatum. Representative image showing probe placement in mouse striatum according to the mouse atlas of Paxinos and Franklin. Intense staining depicts location (arrows) of the probe membrane through the area of striatum. (DOC) [file pone.0022225.s002.doc]
